# Supplementary material for: Pyruvate Dehydrogenase Kinase 1 inhibition mediated oxidative phosphorylation enhancement in cartilage promotes osteoarthritis progression
Source: BMC Musculoskelet Disord. 2023 Jul 20;24:597. doi: 10.1186/s12891-023-06585-6 (PMC10357736; doi:10.1186/s12891-023-06585-6)
Supplement: Supplementary file 1 — Additional file 1. [file 12891_2023_6585_MOESM1_ESM.zip › MF.Gsea.1653627316242/GOMF_CARBON_CARBON_LYASE_ACTIVITY.html]

Details for gene set GOMF\_CARBON\_CARBON\_LYASE\_ACTIVITY[GSEA]

|  || Dataset | 55457\_collapsed\_to\_symbols.cls.cls #OA\_versus\_normal.cls.cls #OA\_versus\_normal\_repos |
| Phenotype | cls.cls#OA\_versus\_normal\_repos |
| Upregulated in class | OA |
| GeneSet | GOMF\_CARBON\_CARBON\_LYASE\_ACTIVITY |
| Enrichment Score (ES) | 0.40503305 |
| Normalized Enrichment Score (NES) | 1.571137 |
| Nominal p-value | 0.014319809 |
| FDR q-value | 0.14265072 |
| FWER p-Value | 0.993 |
Table: GSEA Results Summary

  

Fig 1: Enrichment plot: GOMF\_CARBON\_CARBON\_LYASE\_ACTIVITY      
 Profile of the Running ES Score & Positions of GeneSet Members on the Rank Ordered List

  

| SYMBOL | TITLE | RANK IN GENE LIST | RANK METRIC SCORE | RUNNING ES | CORE ENRICHMENT || 1 | GOT1 | glutamic-oxaloacetic transaminase 1 [Source:HGNC Symbol;Acc:HGNC:4432] | 59 | 0.974 | 0.0613 | Yes |
| 2 | UXS1 | UDP-glucuronate decarboxylase 1 [Source:HGNC Symbol;Acc:HGNC:17729] | 91 | 0.926 | 0.1215 | Yes |
| 3 | GGCX | gamma-glutamyl carboxylase [Source:HGNC Symbol;Acc:HGNC:4247] | 331 | 0.734 | 0.1525 | Yes |
| 4 | TYW1 | tRNA-yW synthesizing protein 1 homolog [Source:HGNC Symbol;Acc:HGNC:25598] | 523 | 0.650 | 0.1816 | Yes |
| 5 | PCK2 | "phosphoenolpyruvate carboxykinase 2, mitochondrial [Source:HGNC Symbol;Acc:HGNC:8725]" | 759 | 0.579 | 0.2024 | Yes |
| 6 | ALDOC | "aldolase, fructose-bisphosphate C [Source:HGNC Symbol;Acc:HGNC:418]" | 870 | 0.551 | 0.2311 | Yes |
| 7 | UROD | uroporphyrinogen decarboxylase [Source:HGNC Symbol;Acc:HGNC:12591] | 905 | 0.541 | 0.2650 | Yes |
| 8 | DERA | deoxyribose-phosphate aldolase [Source:HGNC Symbol;Acc:HGNC:24269] | 975 | 0.523 | 0.2950 | Yes |
| 9 | ARMT1 | acidic residue methyltransferase 1 [Source:HGNC Symbol;Acc:HGNC:17872] | 1080 | 0.500 | 0.3207 | Yes |
| 10 | DDT | D-dopachrome tautomerase [Source:HGNC Symbol;Acc:HGNC:2732] | 1094 | 0.498 | 0.3534 | Yes |
| 11 | NPL | N-acetylneuraminate pyruvate lyase [Source:HGNC Symbol;Acc:HGNC:16781] | 1155 | 0.485 | 0.3815 | Yes |
| 12 | HMGCL | 3-hydroxy-3-methylglutaryl-CoA lyase [Source:HGNC Symbol;Acc:HGNC:5005] | 1703 | 0.398 | 0.3659 | Yes |
| 13 | ME1 | malic enzyme 1 [Source:HGNC Symbol;Acc:HGNC:6983] | 1746 | 0.392 | 0.3891 | Yes |
| 14 | ME2 | malic enzyme 2 [Source:HGNC Symbol;Acc:HGNC:6984] | 1870 | 0.377 | 0.4050 | Yes |
| 15 | PPCDC | phosphopantothenoylcysteine decarboxylase [Source:HGNC Symbol;Acc:HGNC:28107] | 2251 | 0.332 | 0.3979 | No |
| 16 | GAD1 | glutamate decarboxylase 1 [Source:HGNC Symbol;Acc:HGNC:4092] | 2534 | 0.299 | 0.3962 | No |
| 17 | AZIN1 | antizyme inhibitor 1 [Source:HGNC Symbol;Acc:HGNC:16432] | 2786 | 0.271 | 0.3950 | No |
| 18 | UMPS | uridine monophosphate synthetase [Source:HGNC Symbol;Acc:HGNC:12563] | 2936 | 0.257 | 0.4008 | No |
| 19 | SGPL1 | sphingosine-1-phosphate lyase 1 [Source:HGNC Symbol;Acc:HGNC:10817] | 3462 | 0.206 | 0.3739 | No |
| 20 | ALDOA | "aldolase, fructose-bisphosphate A [Source:HGNC Symbol;Acc:HGNC:414]" | 4289 | 0.129 | 0.3183 | No |
| 21 | ME3 | malic enzyme 3 [Source:HGNC Symbol;Acc:HGNC:6985] | 4613 | 0.104 | 0.3002 | No |
| 22 | MLYCD | malonyl-CoA decarboxylase [Source:HGNC Symbol;Acc:HGNC:7150] | 4852 | 0.083 | 0.2874 | No |
| 23 | BCKDHA | branched chain keto acid dehydrogenase E1 subunit alpha [Source:HGNC Symbol;Acc:HGNC:986] | 5671 | 0.018 | 0.2250 | No |
| 24 | ODC1 | ornithine decarboxylase 1 [Source:HGNC Symbol;Acc:HGNC:8109] | 6153 | -0.016 | 0.1887 | No |
| 25 | HDC | histidine decarboxylase [Source:HGNC Symbol;Acc:HGNC:4855] | 6599 | -0.050 | 0.1574 | No |
| 26 | PDXDC1 | pyridoxal dependent decarboxylase domain containing 1 [Source:HGNC Symbol;Acc:HGNC:28995] | 7373 | -0.108 | 0.1046 | No |
| 27 | ECHDC1 | ethylmalonyl-CoA decarboxylase 1 [Source:HGNC Symbol;Acc:HGNC:21489] | 7532 | -0.121 | 0.1005 | No |
| 28 | MOCS1 | molybdenum cofactor synthesis 1 [Source:HGNC Symbol;Acc:HGNC:7190] | 7714 | -0.135 | 0.0956 | No |
| 29 | ALDOB | "aldolase, fructose-bisphosphate B [Source:HGNC Symbol;Acc:HGNC:417]" | 8421 | -0.187 | 0.0533 | No |
| 30 | CRY2 | cryptochrome circadian regulator 2 [Source:HGNC Symbol;Acc:HGNC:2385] | 8536 | -0.196 | 0.0577 | No |
| 31 | DDC | dopa decarboxylase [Source:HGNC Symbol;Acc:HGNC:2719] | 8605 | -0.202 | 0.0660 | No |
| 32 | PCK1 | phosphoenolpyruvate carboxykinase 1 [Source:HGNC Symbol;Acc:HGNC:8724] | 9100 | -0.239 | 0.0438 | No |
| 33 | GAD2 | glutamate decarboxylase 2 [Source:HGNC Symbol;Acc:HGNC:4093] | 9249 | -0.252 | 0.0493 | No |
| 34 | PAICS | phosphoribosylaminoimidazole carboxylase and phosphoribosylaminoimidazolesuccinocarboxamide synthase [Source:HGNC Symbol;Acc:HGNC:8587] | 9475 | -0.272 | 0.0502 | No |
| 35 | AMD1 | adenosylmethionine decarboxylase 1 [Source:HGNC Symbol;Acc:HGNC:457] | 10415 | -0.363 | 0.0017 | No |
| 36 | MVD | mevalonate diphosphate decarboxylase [Source:HGNC Symbol;Acc:HGNC:7529] | 10934 | -0.423 | -0.0100 | No |
| 37 | CRY1 | cryptochrome circadian regulator 1 [Source:HGNC Symbol;Acc:HGNC:2384] | 11247 | -0.463 | -0.0029 | No |
| 38 | CSAD | cysteine sulfinic acid decarboxylase [Source:HGNC Symbol;Acc:HGNC:18966] | 11649 | -0.525 | 0.0013 | No |
| 39 | CYP17A1 | cytochrome P450 family 17 subfamily A member 1 [Source:HGNC Symbol;Acc:HGNC:2593] | 12154 | -0.632 | 0.0049 | No |
| 40 | PISD | phosphatidylserine decarboxylase [Source:HGNC Symbol;Acc:HGNC:8999] | 12581 | -0.785 | 0.0248 | No |
Table: GSEA details [plain text format]

  

Fig 2: GOMF\_CARBON\_CARBON\_LYASE\_ACTIVITY      
 Blue-Pink O' Gram in the Space of the Analyzed GeneSet

  

Fig 3: GOMF\_CARBON\_CARBON\_LYASE\_ACTIVITY: Random ES distribution      
 Gene set null distribution of ES for **GOMF\_CARBON\_CARBON\_LYASE\_ACTIVITY**
